# Isolation and Culture of Post-Natal Mouse Cerebellar Granule Neuron Progenitor Cells and Neurons 

**DOI:** 10.3791/990

**Published:** 2009-01-16

**Authors:** Hae Young Lee, Lloyd A. Greene, Carol A. Mason, M. Chiara Manzini

**Affiliations:** Department of Genetics and Development, Columbia University; Department of Pathology and Cell Biology, Columbia University; Department of Neuroscience, Columbia University; Department of Neurology, Beth Israel Deaconess Medical Center, Harvard Medical School

## Abstract

The cerebellar cortex is a well described structure that provides unique opportunities for studying neuronal properties and development^1,2^. Of the cerebellar neuronal types (granule cells, Purkinje cells and inhibitory interneurons), granule neurons are by far the most numerous and are the most abundant type of neurons in the mammalian brain. In rodents, cerebellar granule neurons are generated during the first two post-natal weeks from progenitor cells in the outermost layer of the cerebellar cortex, the external granule layer (EGL). The protocol presented here describes techniques to enrich and culture granule neurons and their progenitor cells from post-natal mouse cerebellum. We will describe procedures to obtain cultures of increasing purity^3,4,^ which can be used to study the differentiation of proliferating progenitor cells into granule neurons^5,6^. Once the progenitor cells differentiate, the cultures also provide a homogenous population of granule neurons for experimental manipulation and characterization of phenomena such as synaptogenesis, glutamate receptor function^7^, interaction with other purified cerebellar cells^8,9^ or cell death^7^.

**Figure:**
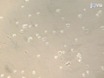


## Protocol

### Part 1: Setting up (1-2 days before dissection) (Not shown on video)

PREPARING CULTURE SOLUTIONS AND MEDIA:
  4X CMF-PBS-EDTA (calcium and magnesium free-phosphate buffered saline-EDTA for Percoll dilutions): per liter, add 32 g NaCl, 1.2 g KCl, 8 g glucose, 2 g NaH_2_PO_4_, 1 g KH_2_PO_4_, 8 ml 2% NaHCO_3_ stock, 10 ml 1M EDTA (pH 8.0) to distilled/deionized water. Adjust the volume to 1 liter and the pH to 7.4. Filter sterilize.
  HBSS-GLUCOSE: Add glucose (6 grams/liter) to calcium and magnesium free Hank’s Balance Salt Solution (HBSS) and sterile filter. 500 ml can be stored at 4°C for up to a month.
  CULTURE MEDIA: serum-free medium (SFM) and 10% FBS medium. To 48 ml of Neurobasal A-Medium add 500 μl 100X GlutaMAX I, 500 μl 100X Penicillin-Streptomycin (final 100 Units penicillin and 100 μg streptomycin), 6.25 μl 2 M KCl (final 250 μM). Split in 2 aliquots of 9 ml and 40 ml. To prepare 10% FBS medium, add 1ml of heat-inactivated FBS to the 9 ml aliquot. To prepare SFM, add 800 μl of the serum-free supplement B-27 to the 40 ml aliquot. Filter sterilize and store at 4°C for a maximum of 2 weeks. For best results make fresh media for each experiment.
PREPARING PERCOLL DILUTIONS :
  Percoll is supplied as a liquid and it must be acidified before use. To prepare the stock, add 1N HCl little by little to the Percoll solution over a 1-2 hour period until pH 7.4 is reached. Adding the HCl too rapidly will cause the Percoll to aggregate. Approximately 6.5ml of 1N HCl are needed for each liter of Percoll. Filter sterilize and store at 4°C.
  A 35% and a 60% (vol:vol) Percoll dilution will be used in the Percoll gradient step. Prepare 100 ml Percoll dilutions containing 35 or 60 ml of Percoll stock, 25 ml of 4X CMF-PBS-EDTA and distilled/deionized H2O to the final volume. Add a few drops of toluidine blue to the 60% solution; it will help visualize the interface and the cells. Filter sterilize and store at 4°C.
  4X CMF-PBS-EDTA should not be older than 3 months, as inadequate buffering will cause increased cell death during the procedure.
PREPARING COVERSLIPS: Glass coverslips (best if from Carolina Biological Supply Company) are washed with 10% HCl overnight at room temperature with mild agitation, rinsed thoroughly with distilled/deonized water and stored in 70% ethanol. Before use, single coverslips are flame-sterilized by briefly holding them with forceps over an open flame (Bunsen burner) until the ethanol evaporates. 12-mm glass coverslips can be placed in 4-well culture plates and 25-mm coverslips in 6-well culture plates.
COATING CULTURE VESSELS WITH SUBSTRATE: For immunofluorescence staining or drug treatments glass coverslips are more appropriate since they can be easily mounted on glass slides for visualization under a microscope. When large numbers of cells are needed for collecting protein or RNA samples, cells can be cultured on plastic culture dishes.The night before dissection, glass coverslips or plastic dishes for culture must be coated with poly-D-lysine (500 μg/ml; 800 μl/well for 6-well plates or 350 μl/well for 4-well plates). 5 mg/ml stock solution of poly-D-lysine is stored at -20°C, then diluted in sterile distilled/deonized water and sterile filtered right before use. Culture vessels are incubated at 37°C overnight (or for at least for 2 hrs before plating) and washed twice with sterile distilled/deonized water right before plating.
PREPARING PREPLATING DISHES: These dishes will be used for pre-plating the isolated cerebellar cells before culture to remove glial contaminants, which adhere more readily to a lower concentration of substrate. Coat two 60-mm plastic culture dishes with 4 ml per dish of poly-D-lysine (100 μg/ml; Note this is 1/5 of the concentration used for culturing). Incubate at 37°C overnight (or for at least for 2 hrs before dissection). Right before the dissection, remove the poly-D-lysine solution, wash the dishes twice with sterile water and allow to dry in the hood.
Sterilize dissection tools in the autoclave or on the day of dissection, immerse the tools in 70% ethanol for 20 minutes. Required tools: Permaset scissors, microdissecting scissors, four Dumont #5 dissecting forceps.

### Part 2: Preparing for Dissection (day of dissection) – (Demonstrated in video)

All of the following procedures are performed in a tissue culture hood unless noted.

Wipe down the dissecting area with 70% ethanol. Warm the media to 37°C in a water bath or in a 5% CO_2_ 37°C incubator.
When starting a new Papain Dissociation System Kit (Worthington), prepare the solution of albumin-ovomucoid inhibitor. Add 32 ml of EBSS (Earle’s Balanced Salt Solution; provided in the kit) to the albumin-ovomucoid inhibitor mixture and allow the contents to dissolve while preparing the other components. Reconstitute for the first use, then store the remaining solution at 2-8°C and use until the five isolations allowed by each kit are completed.
Add 5 ml of EBSS to one papain vial from the Dissociation Kit (each vial is sufficient for dissociation of 4 to 15 cerebella from postnatal day 5 mice). Place the papain vial in a 5% CO_2_ 37°C incubator or 37°C water bath for 5 to 10 minutes until the papain is completely dissolved and the solution appears clear. Maintain the solution at room temperature during dissection.
Add 500 μl of EBSS to one DNase vial from the Dissociation Kit. Mix gently by tapping the vial since DNase is sensitive to shear denaturation. Add 250 μl of this solution to the vial containing the papain (final concentration is 20 units/ml papain and 0.005% DNase). Save the remaining DNase vial for use in step 5.1 or 6.1.

### Part 3: Dissection and Meninges Removal

The optimal age for granule cell culture from C57BL/6J mice is postnatal day 4-6, when the number of granule cell progenitors in the EGL peaks1,10. Dissect pups one at a time and as you become more familiar with the cerebellar dissection try to reduce dissection time to no more than 6 minutes per pup. Speed is of the essence. If the Percoll gradient step is bypassed, eight postnatal-day-5 mouse pups can yield enough cells for one 6-well culture plate (or six 25-mm coverslips) or for six 4-well culture plates (or twenty-four 12-mm coverslips). Approximate yield is 4 to 5x106 cells per pup and plating densities can range between 1 and 5X105 cells/cm2. Since 15-20% cells are lost during the Percoll step4, more animals are needed to obtain the same desired cell density.
Pipette 15 ml of HBSS-glucose into a 60-mm plastic tissue culture dish and 10 ml into a sterile Falcon 15 ml conical plastic tube. Put on ice.
Outside the hood, wipe the head of the pup with 70% ethanol. Use Permaset scissors to decapitate the pup. (Decapitation will not be shown in the video.)
In the hood, hold the head with Dumont forceps so that you can clearly see the back of the skull. Access the brain by inserting microdissecting scissors into the foramen magnum and cutting straight toward the eyes. Using forceps, peel away the skin and lift up the skull to expose the brain. Using Dumont forceps, pinch off the cerebellum and the surrounding midbrain and transfer it to the dish with HBSS-glucose. (It is easier to manipulate the cerebellum and remove the meninges if the cerebellum is still attached to the surrounding tissue).
Under the dissecting microscope, gently peel the meninges off the cerebellum and also between the lobes with one fine Dumont #5 forceps while using the other forceps to anchor down the cerebellum to the plate. You will notice blood vessels on the surface of the cerebellum. These blood vessels are a good way to identify the meninges. Remove the meninges until the cerebellum takes on a matted white appearance. Separate the cerebellum from the rest of the midbrain. Turn it to its ventral side and remove the choroid plexus, which looks like a reddish ribbon between the ventral cerebellum and adjacent midbrain. Place each cerebellum in cold HBSS-glucose in a 15 ml conical tube on ice as soon as dissected. Change the dissecting solution to fresh HBSS-glucose after dissecting 3 brains.

### Part 4: Cell suspension

Once all the dissections are finished, remove the HBSS-glucose from the tube and replace it with the papain solution made in step 2.4. Although the kit recommends cutting the tissue in small pieces, we find that it is not necessary to cut or mince the cerebella at this age. Place the tissue + papain solution (in a 15 ml conical tube) in a 37°C water bath or 5% CO_2_ 37°C incubator for 15 to 20 minutes. For 4 to 8 cerebella, 15 minutes at 37°C is sufficient; for a larger number of cerebella 20 to 30 minutes is better. Gently agitate the tube every 3 to 4 minutes.
Triturate the mixture with a sterile p1000 aerosol pipette tip that has been coated with FBS. Do this step gently to prevent any air bubble formation. Fire polished glass pipettes can be used, but we find that serum coated sterile p1000 aerosol tips work just as well. To coat the pipette tip with FBS, pipette FBS up and down twice just before use. About 10 up and down movements with the pipette are enough to dissociate the tissue. The solution will become cloudy. Allow the suspension to sit for 30-60 seconds so that any large pieces of undissociated tissue will settle to the bottom of the tube.
Remove the suspended cells (careful not to remove the undissociated tissue pieces at the bottom) into a fresh 15 ml conical tube with a serum coated pipette tip. Centrifuge at approximately 200xg for 5 minutes at room temperature. Prepare re-suspension medium. To do this, mix 2.7 ml EBSS with 300 μl of reconstituted albumin-ovomucoid inhibitor solution in a sterile tube. Add 150 μl of DNase solution from step 2.4.
After centrifuging, discard the supernatant and immediately resuspend the cell pellet in the diluted DNase/albumin inhibitor solution prepared in step 4.3 with a serum coated p1000 aerosol pipette tip.
Prepare a discontinuous density gradient as follows. Add 5 ml of albumin-ovomucoid inhibitor solution to a 15 ml conical tube. Carefully layer the cell suspension on top. Centrifuge at approximately 70xg for 6 minutes at room temperature. Dissociated cells pellet at the bottom of the tube, membrane fragments remain at the interface.
If you wish to obtain cultures that are enriched in granule cells, bypass the Percoll gradient separation step (which yields purer cultures of cerebellar granule cells), and proceed to Part 6. If you wish to further isolate the granule cells from glia and large interneurons by Percoll gradient separation, proceed to Part 5. Use of the Percoll gradient step will reduce the yield of cells. In our hands, the reduction in yield is approximately 20-35%. However, there is an increase in the enrichment of granule neurons and progenitor cells from approximately 90% to 95-99%.

### Part 5: Percoll Gradient Separation

Discard the supernatant and immediately resuspend the pellet in 2 ml of HBSS-glucose with 50 μl of DNase from step 2.4. Filter the cell suspension though a nylon mesh (pore size 70 μm) by gravity. This step will remove large non-neuronal cells and provides for a better single cell suspension.
Prepare two fire-polished glass Pasteur pipettes. To do this, gently flame the tip of a glass pipette until the edge is smoothened and the pipette bore is 40-50% of the original size. Take care not to make the opening too small.
Prepare Percoll gradient. Place 10 ml of 35% Percoll solution in a 50 ml sterile conical tube. 60% Percoll (10 ml) is loaded into a 10 ml sterile syringe with a sterile spinal needle attached. Gently layer the 60% Percoll solution underneath the 35% solution, by adding the 60% solution at the bottom of the tube with the spinal needle. Take care to keep a sharp interface between the two layers. Add the cells to the top of the gradient using a fire-polished pipette, gently adding them along the side of the tube without disturbing the interface.
Carefully place the tube in the centrifuge and centrifuge at 1800xg. Ramp up the speed one step every 20 seconds (increments of speed are as follows: approximately 18, 70, 200, 440, 850, 1100, 1800xg) so that it takes approximately 2 minutes to reach the desired speed. Start the timer to 10 min when 1800xg is reached. When finished, decrease speed gradually one step every 20 seconds.
Remove the upper interface of the gradient (containing large glia, Purkinje cells, and interneurons) and discard.
Carefully remove the cells at the interface between the 35% and 60% Percoll with a fire-polished pipette and transfer to a 50 ml conical tube. Resuspend in 3 volumes of HBSS-glucose and mix by inverting several times. Centrifuge for 5 minutes at 1100xg.
Remove the supernatant and add 4 ml of 10% FBS medium with 50 μl of DNase. Resuspend the pellet gently using a fire-polished pipette to form a single cell suspension. Proceed to step 6.2.

### Part 6: Pre-plating and Plating

Discard the supernatant and immediately resuspend the pellet in 4 ml of 10% FBS medium with 50 μl of DNase from step 2.4. Filter the cell suspension though a nylon mesh (pore size 70 μm) by gravity. This step will remove large non-neuronal cells and provides for a better single cell suspension.
Plate the collected cells on a poly-D-lysine coated pre-plating dish for 20 minutes in a 5% CO_2_ 37°C incubator. Repeat with a fresh dish. The astroglia and heavier cells will settle down and adhere to the dishes and leave the small granule neurons and neuron progenitor cells floating. After the incubation, loosely adhered granule neurons and neuron progenitor cells are easily dislodged and loosened with gentle tapping of the plate.
Collect the granule neurons and neuron progenitor cells in a 15 ml conical tube and centrifuge at 200xg for 5 minutes. Resuspend the pellet in 1 ml of serum-free medium and count an aliquot of 10 μl using a hemocytometer.
Add serum-free medium to reach the desired cell density. Rough guidelines to numbers of cells to plate for medium density: for 6-well plates, 3.5 to 4 million cells; for 4-well plates, 650,000 cells. For 6-well culture plates, I plate 1.5 ml /well and for 4 well-plates, I plate 0.5 ml/well. The cells are maintained in a 5% CO_2_ 37°C incubator. Change the medium completely 24 hours later with subsequent changes every two to three days.

## Discussion

This protocol is based on modifications of procedures that have been described in the past^3,7,11^. There are several important points to note as discussed below.

The granule neurons and progenitor cells adhere within 2 hours of plating. Healthy cells have a round morphology under the phase-contrast microscope^4^. Within 24 hours after plating, healthy cells will spread evenly around the coverslips or the plastic well and will form processes. At the time of the first medium change, after 24 hours, there will be a few dead floating cells. This is normal, as a few cells will die or become unhealthy during the dissociation process. However, if after 48 hours the cells are clumped together with varicosities on their processes, either the cells are unhealthy or the poly-D-lysine substrate is toxic. As with most substrates, the efficacy of poly-D-lysine is lot dependent and each new lot must be tested. Images of healthy cultures can be found in references ^4,11,^ and ^20^. Healthy cells can be maintained in culture for up to two weeks^8^.

Granule neuron progenitors begin to differentiate upon plating^8,12,13,14^. Once the optimal plating density is established for your studies, it should be maintained, because proliferation is promoted by factors such as Notch signaling^15^, and cells will proliferate more at higher densities. The proliferation of granule neuron progenitors can be substantially prolonged by adding sonic hedgehog (Shh) to the medium^12,13,14^. Laminin can also be added as a substrate in addition to poly-D-lysine to promote neurite outgrowth^16,17^. These features of the granule cell culture offer a system for studying either the biology of granule neurons or the regulation of differentiation of progenitors cells into neurons^6,18,19^.

Isolated cerebellar cells from postnatal mice are initially comprised of a mixture of granule neurons and granule neuron progenitors at different stages of the cell cycle^8^. There are also astroglia and interneurons present in the isolation/culture and further purification of granule neurons and granule progenitors (95%-99%) is attained by the Percoll gradient separation^4^. Enriched granule cells obtained without the use of the Percoll gradient separation step have been used to study the regulation of proliferation and differentiation of granule neuron progenitors, ^18,19,20^. In corroboration of this, we find that cells in the population isolated by bypassing the Percoll gradient separation step respond robustly to Shh by remaining in the cell cycle for several days, and that without Shh addition, there is very little proliferation in the cultures as indicated by staining with the cell proliferation maker, Ki67. However, if the cultures are to be used beyond several days *in vitro*, it is recommended that the Percoll gradient step be included to decrease contamination of the granule neurons by proliferating non-neuronal cells.
